# Devising Digital Twins DNA Paradigm for Modeling ISO-Based City Services

**DOI:** 10.3390/s21041047

**Published:** 2021-02-04

**Authors:** Hawazin Faiz Badawi, Fedwa Laamarti, Abdulmotaleb El Saddik

**Affiliations:** 1 Multimedia Communications Research Laboratory (MCRLab), School of Electrical Engineering and Computer Science, University of Ottawa, Ottawa, ON K1N 6N5, Canada; flaamart@uOttawa.ca (F.L.); elsaddik@uottawa.ca (A.E.S.); 2 Department of Computer Science, College of Computer and Information Systems, Umm Al-Qura University, Mecca 24381, Saudi Arabia

**Keywords:** digital twin, DNA, smart health, well-being, standards, ISO 37120, data analysis, artificial intelligence

## Abstract

Digital twins (DTs) technology has recently gained attention within the research community due to its potential to help build sustainable smart cities. However, there is a gap in the literature: currently no unified model for city services has been proposed that can guarantee interoperability across cities, capture each city’s unique characteristics, and act as a base for modeling digital twins. This research aims to fill that gap. In this work, we propose the DT-DNA model in which we design a city services digital twin, with the goal of reflecting the real state of development of a city’s services towards enhancing its citizens’ quality of life (QoL). As it was designed using ISO 37120, one of the leading international standards for city services, the model guarantees interoperability and allows for easy comparison of services within and across cities. In order to test our model, we built DT-DNA sequences of services in both Quebec City and Boston and then used a DNA alignment tool to determine the matching percentage between them. Results show that the DT-DNA sequences of services in both cities are 46.5% identical. Ground truth comparisons show a similar result, which provides a preliminary proof-of-concept for the applicability of the proposed model and framework. These results also imply that one city performs better than the other. Therefore, we propose an algorithm to compare cities based on the proposed DT-DNA and, using Boston and Quebec City as a case study, demonstrate that Boston has better services towards enhancing QoL for its citizens.

## 1. Introduction

Digital twins (DTs) technology has come a long way in industry since it was first developed by the National Aeronautics and Space Administration (NASA) to mirror the state of health of the flying twin [1] and coined as a core concept for the industrial future by Vickers and Grieves in 2002 [2]. However, the extensive deployment of digital twin technology remained specific to the industrial field, which is definitely closer to machines and physical systems, with a paucity of research in academia for deployment in other non-industrial fields closer to humans, such as health, environment and recreation. Despite the announcement of digital twins as the fifth technology in the top 10 strategic technology trends for 2017 by Gartner [3], the fourth in the same list in 2018 [4] and 2019 [5], and the fifth trend in The Gartner Hype Cycle for Emerging Technologies published in August 2020 [6], few researchers recognized its potential role outside of the industrial field. The research in [7] is one of the leading works that promoted the vision of applying digital twins technology beyond manufacturing, to purposes such as enhancing human well-being and improving quality of life (QoL) in smart cities, thereby expanding the original definition of digital twins. The incorporation of living and non-living physical entities into the definition of digital twins [7] introduced the seemingly unlimited benefits of using the technology to monitor, understand, and optimize the functions of city services aimed at improving the quality of life and well-being of citizens. This expanded vision is of particular interest given that the percentage of the world population living in cities is expected to rise to 68% by 2050 [8], which emphasizes the importance of efficient management of city services [9]. 

However, there is a gap in the literature: currently no unified model for city services has been proposed that can guarantee interoperability across cities, capture each city’s unique characteristics, and act as a base for modeling digital twins. A general, standardized model such as this would need to meet the following requirements:

- *Unified and unique*, accounting for all city services while protecting the unique identity of each city and providing the means to model special services (being unified implies that the model also needs to be standardized to guarantee interoperability);
- *Entity-inclusive,* taking into account human such as citizens, stakeholders and regulatory authorities-due to the central role of users contribution in the provision of smart city services [10], in addition to other entities such as IoT entities early on in the analysis and design stages, to identify correlations between them and city services based on the expanded definition of DTs that incorporates living and non-living physical entities into the definition of DTs [7];
- *Contextualized and customizable*, tracking the dynamic state of the city by collecting geo-temporal data as well as emergent data from interactions between citizens and city services;
- *Visualizable*, providing visual representations of results and analysis to citizens, stakeholders and regulatory authorities who care about the performance and sustainability of city services and the enhancement of those services for improved quality of life.

These requirements need to be achieved based on recognized international standards of city services in order to fulfill the interoperability need. ISO 37120 [11] is one of the leading standards that serves this purpose as it was developed with the sustainable development of communities in mind, to provide a set of indicators for measuring the performance of city services and improved quality of life. 

In this paper, we propose a digital twin DNA (DT-DNA) model to build a digital twin of city services based on ISO 37120. We also propose a framework that shows how the built DT-DNA can be utilized in different analytics to provide insight into the state of city services, particularly health services.

We leverage the DNA double helix model in the domain of digital twins to model DT-DNAs of city services. The proposed model can uniquely identify a DT-DNA for each city service among all other services, even those of the same type, in a similar way to DNA, which captures the unique identity of each organism among all other organisms. For example, each health service will have its own DT, uniquely identified among multiple health services in the city, using the proposed DT-DNA model. 

We believe that the ability of the DNA to protect the genetic blueprint of human and other organisms throughout history can be mimicked and applied to the modeling of city services. The characteristics that justify our choice to build DT-DNA on the analogy of the biological DNA will be discussed in a subsequent section.

The rest of this paper is organized as follows: Section 2 presents background information and related work. Section 3 provides the analysis of the DNA biological model and smart cities, and proposes the four bases of DT-DNA. Section 4 discusses the proposed DT-DNA model and framework to build DTs of city services using indicator values based on ISO 37120. A proof-of-concept using standardized data and the results are presented in Section 5. Further discussion on the modeling results is presented in Section 6 and Section 7 highlights future work and concludes this paper.

## 2. Background and Related Work

In this section, we start by giving an overview of background concepts on which we based our work, namely, digital twins, DNA, and the ISO 37120 standard. Then, we present an overview of existing literature on leveraging digital twins technology for city services. 

### 2.1. Background

#### 2.1.1. Digital Twins (DTs)

DTs research recently gained attention in academia for non-industrial deployment of this technology in fields that are closer to humans such as health, environment and recreation. According to [7], DTs are defined as “digital replications of living as well as non-living entities that enable data to be seamlessly transmitted between the physical and virtual worlds. Digital twins facilitate the means to monitor, understand, and optimize the functions of all physical entities and for humans provide continuous feedback to improve quality of life and well-being.”

Although DTs technology is currently a hot topic in academia, and a top priority for several industries, a generally accepted model for DTs of city services does not yet exist. Reviewing literature shows that the DT concept is already in use in several applications areas but many challenges have to be addressed and interoperability is one of them [12]. 

Cities comprise multifaceted services and domains such as health, transportation and environment that must function together. Since cities have their own characteristics and needs, the challenge is to define a model of city services that is general enough to evaluate the performance of services that exist in all cities, but flexible enough to capture and evaluate the performance of unique services that exist in each city. Thus, we suggest building a DT model of city services that encompasses different tasks, contexts and stakeholders based on ISO-standardized services. In this way, all cities can share a generalized DT model. However, each city will have its own unique DT that can be customized by multi-source, dynamic values for each parameter. This will facilitate building DTs that are able to represent a diverse range of city services. This design is fairly analogous to various organisms, where each is created to serve a specific purpose while also sharing a general model—the DNA within their cells [13]. DNA contains the instructions, called genetic fingerprints, that make each organism unique [14]. In the following section, we provide an overview of DNA to clarify the purpose of using this analogy in the proposed DT-DNA paradigm. We justify the purpose of discussing the anatomy of smart cities in Section 3.

#### 2.1.2. DNA

**Figure 1:**
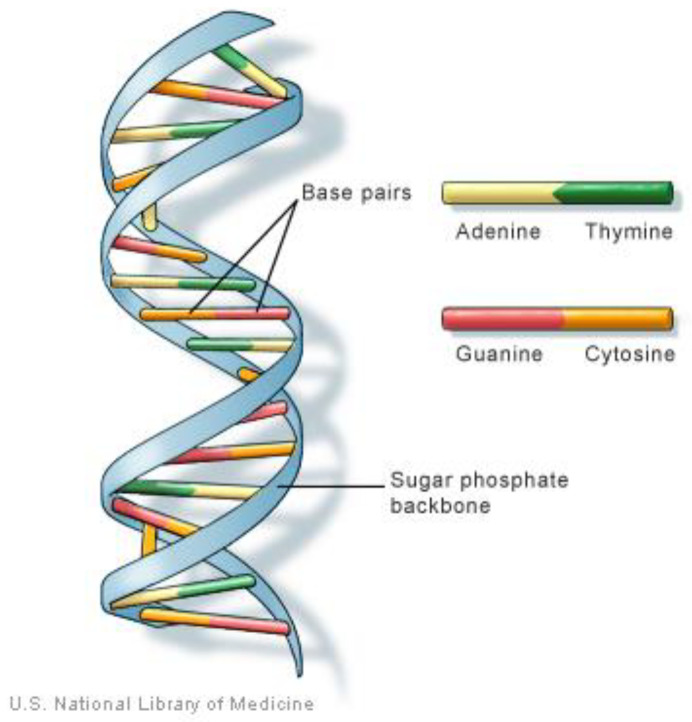
DNA model in biology [16].

DNA (deoxyribonucleic acid) molecules are found inside a cell’s nucleus and each molecule is tightly packaged in a form called a chromosome [14]. Genes are specific sections of the chromosome that contain the instructions to produce proteins, which do most of the work in our bodies [14]. Genes represent 1% of the DNA sequence in the chromosome and the remainder of this sequence controls the time, the amount, and the method of protein production in the cell [14]. Each organism has a specific number of chromosomes that distinguishes it from others, but all share the unique double helix structure of a DNA molecule proposed by Watson and Crick [15]. The genetic instructions are stored as a code made up of four chemical bases: adenine (A), guanine (G), cytosine (C), and thymine (T), which pair up to form units called base pairs, where A pairs with T and C pairs with G, and then arrange into the two long strands that form the double helix [16] depicted in Figure 1. These bases are ordered in a specific sequence to determine the information available for building and maintaining an organism, similar to sequencing letters of the alphabet in a certain order to form words and sentences [16]. Each human contains about 3 billion base pairs and 20,000 genes [14]. Approximately 99.9 % of these base pairs are the same in all people, while 0.1% are variants that differentiate humans and make everyone unique [16]. Each cell in the human body contains 23 pairs of chromosomes. Of these, 22 pairs are identical in both males and females and the 23rd pair is the one that distinguishes them. The double helix structure of DNA is the unique shared model among organisms, but the sequential order of the DNA base pairs along its sides is the distinguishing factor.

#### 2.1.3. ISO 37120

This standard, which is officially called “ISO 37120: Sustainable cities and communities–Indicators for city services and quality of life,” is the leading international standard on city indicators, developed by the ISO sustainable cities and communities technical committee [11].

ISO 37120 was developed as a tool for city stakeholders to facilitate the implementation of policies and theoretical plans designed to promote sustainable and livable cities and enhance quality of life for all citizens. Since existing indicators are often not standardized, consistent, or comparable over time or across cities, the ISO 37120 technical committee establishes a uniform approach to measuring the performance of city services and quality of life. There are 17 themes or services for ISO 37120 and approximately 100 indicators, either core or supportive, categorized under the themes to assess the performance of various services in a city.

### 2.2. Digital Twins for City Services

In this section, we start with an overview of the use of digital twins technology for city services. We then focus on health as an area of city services that can leverage DTs technology to enhance citizens’ quality of life. 

Conducting a search on Scopus by running the following query results in 23 documents:

TITLE-ABS-KEY ((“city” OR “cities “) AND (“service” OR “services”) AND (“Digital Twin” OR “Digital Twins”))

Analyzing these search results reflects recent interest in utilizing digital twins technology in various city services in areas such as health and well-being [17], evaluation of carbon emissions [18], comfort monitoring in universities campuses [19] traffic monitoring [20], and infrastructure [21]. 

Health and well-being play a prominent role in enhancing quality of life [22], hence health is one of the main service areas targeted by research to leverage digital twins technology. DTwins Ecosystem for Health and Well-being [23] was one of the first research initiatives to introduce the utilization of DTs technology in the health and well-being field. Based on this ecosystem, research in [17] proposed an ISO/IEEE 11073 standardized digital twins framework architecture for health and well-being, geared towards fostering the use of healthcare services in smart cities. It also emphasized the importance of standardization to guarantee interoperability of the developed digital twins. ISO/IEEE 11073 [24] is a family of standards developed for personal health devices to facilitate the collecting of health data by these devices. A recent literature review on systems compliant with ISO/IEEE11073 is presented in [25] and the standardization of a shoe insole based on this family of standards is presented in [26]. 

## 3. Digital Twins DNA Model: Requirements Analysis

In this section we present an analysis of the biological DNA model and highlight its features, which provide the building blocks for our DT-DNA model. Following this (in Section 3.2), we present an analysis of the influencing factors of smart cities modeling, in order to determine the main bases of our DT-DNA model of city services.

### 3.1. DNA Model

The ability of DNA to represent and protect the genetic blueprint of human and other organisms throughout history is what motivated us to mimic and utilize this model for modeling DTs of city services. To deliver effective services to citizens, a mechanism is needed to handle various kinds of data collected from heterogeneous devices and applications. As discussed previously, the genes sequence contains instructions to produce proteins, whereas the rest of the sequence regulates the time, the amount, and the method of protein production in the cell [14]. The biological DNA model is characterized by many features including the following, which motivated us to consider this analogy:

- Unified Model: all chromosomes or DNA sequences share a common structure, which is the double helix DNA with four bases: A, T, C, and G and the sequential order of these bases causes the difference in the chromosomes’ values.
- Unique Model: all chromosomes are identical in the same organism (all cells have an identical copy and number of DNA) but differ from one organism to another. Thus, all humans have the same number of chromosomes, but each human has a unique genetic fingerprint and variance.

The same analogy can be made for city services. Any robust model of city services must be unified and unique, similar to the way in which DNA captures the unique identity of each organism using a common structure.

### 3.2. Anatomy of Smart Cities 

In order to successfully design a digital twins model for city services, we identified four essential requirements outlined in Section 1. We argued that any unified model that could work as a base for building digital twins for city services would need to be unified and unique, entity-inclusive, contextualized and customizable, and visualizable. Using the DNA analogy as a starting point and incorporating ISO 37120 standards [11], we were able to meet the first of these requirements: design a model that is unified and unique.

Our next step involved analyzing the literature on the anatomy of cities in order to identify other influencing factors that shape city services. 

In our analysis of the literature, humans are identified as one of the influencing factors [10,27]–citizens who are the targets of services, and stakeholders and authorities who regulate services–consistent with our *entity-inclusive* requirement outlined in Section 1, in terms of human inclusion. Indeed, many studies emphasize the role of the human factor in smart cities such as citizens’ contribution in the provision of services through providing data [10], and citizens’ engagement in the validation of the provided services [27]. In terms of entities other than human, the Internet of Things (IoT) is the backbone of smart cities according to [28], hence all its four components: the things, the local area network, the internet and the cloud are considered to be influencing factors on the smart cities and their services. Automated analysis of the collected data from these components will be performed in data analysis module using machine and deep learning algorithms as shown in Section 4.2.

We also identified tasks included in each service as another influencing factor shaping city services. ISO 37120 [11] fulfills the operability requirement and the set of city services along with indicators to evaluate each city’s performance and its citizens’ quality of life. In addition, various contextual data are considered influencing factors. These factors are consistent with the *contextualized and customizable* requirement outlined in Section 1. The literature highlights the important role of geographical data in smart cities modeling. Spatial data acts as a link between authoritative spatial information used by governments, and voluntary information, crowd-sourced from various distributed sources in smart cities [29]. This justifies the linkage between the geographical data and context components in the proposed model, as shown in the next section. 

Based on our analysis of the literature on the anatomy of smart cities, we identified four main components that shape city services:

- Tasks, included in different types of services—each service consists of a set of tasks that defines the purpose of the provided service.
- Included entity in a given service, which ranges between humans and any other entity in the IoT of the smart city. Human represent different members of urban societies, including citizens; stakeholders such as managers, politicians, researchers, business leaders, planners, designers; and authorities regulating the provision of services. IoT objects represent the physical entities or things that enable the provision of different smart services in agriculture, health care, mobility, security and surveillance, energy, and building management fields.
- Context, which represents contextual data collected from various sources as defined by [30], including “any information that can be used to characterize the situation of an entity.” The sources may be hard sensors, such as those distributed around the city as part of the smart grid, e.g., sensors embedded in city streets for traffic; or soft sensors, such as software applications used for ride-sharing as example of smart mobility, watering of farms as example of smart farming, telemedicine as example of smart health, and determining an individual’s geographic location as example of smart tracking.
- Geographical data, which represents the spatial and temporal data of the cities and entities that play an essential role in the provided services in the city.

In this way, and building on our DNA analogy, we were able to define the fundamental bases for our proposed DT-DNA model, as described in Section 4.1.

## 4. Proposed Model and Framework

The DT-DNA model proposed in this paper considers various aspects of city services, including service type, tasks, service provision contexts, spatiotemporal data, involved stakeholders and authorities, and citizens who are users of city services. To meet the requirements listed in the introduction, we designed the proposed DT-DNA model to handle data coming from various sources as standardized or non-standardized by developing an ISO 37120 wrapper. This approach allows the DT-DNA model to accommodate valuable city data that do not adhere to the ISO standard (i.e., non-standardized data) but are nevertheless important aspects of a city’s services.

Consistent with the analysis presented above, DT-DNA share the same structure but differ in the values of the services provided—similar to biology where all cells share a common DNA structure but differ in the order values of the four bases in their sequences.

Based on the fact that each human cell contains 23 chromosomes that define the genetic properties of the organism [31], we suggest considering each city as a cell that contains 23 chromosomes. Each chromosome represents a service provided by the city. Of these, 17 are chromosomes based on ISO 37120, which defines 17 standardized services [32]: economy, education, energy, environment, recreation, safety, shelter, solid waste, telecommunication and innovation, finance, Fire and emergency response, governance, health, transportation, urban planning, wastewater, and water and sanitation The remaining 6 chromosomes may accommodate new services proposed in future versions of ISO 37120, as well as unique, city-specific services. The proposed DT-DNA model and framework have been designed to facilitate comparisons of services within and between cities and across time, based on availability of data.

### 4.1. Proposed DT-DNA Model

In keeping with our DNA analogy and building on our analysis of the literature presented in Section 3.2, we have identified four bases, A, T, G, and C for our DT-DNA model. We will start by describing our analogous G and C bases, followed by A and T bases. Each base is defined by a set of identifiers, as shown below, and we adopt a “fixed-location” characteristic for these identifiers, maintaining the same location for each, represented by a 2-alphabet code in the DT-DNA strand.

- **Geo-temporal (G):**

The G base in the proposed DT-DNA model represents the geographical and temporal information of the city where the intended service is provided. Geo-temporal information plays a critical role for both real and digital twins [33]. We define this base by four main identifiers described in the following:

***Continent-Country-City-Year***

**Continent**: This identifier represents the code of the continent where the city is located. We used a 2-alphabet continent code associated with the list of ISO 3166 Country Codes [34]. The complete list of the seven continents is shown in Table 1. 

**Country**: This identifier represents the code of the country where the city is located. We used the complete list of ISO 3166 Country Codes [34], which corresponds to the list of top-level domains (TLDs) of all countries that is developed and maintained by the Internet Assigned Numbers Authority (IANA) [35]. 

**City:** This identifier represents the city in which the intended service will be provided. A 2-alphabet code derived from the city name is used for this purpose. The latitudinal and longitudinal coordinates of the city could also be used here.

**Year**: This identifier represents the code for the year in which the DNA of the city services DT is built.

- **Context (C):**

The C base in the proposed DT-DNA model represents the context of the city services DT. In the proposed model, the context is defined by any source of information surrounding a provided service that influences its status. The city context is defined by the environmental context (EC) according to the ISO 37120 standards [11], which we adopt on a city level in the proof-of-concept described in Section 5. Environmental context is determined by: the city’s surface (S), either flat (F) or hilly (H), or both; location (L), either sea (S) or inland (N); and kind of rock it is built on (R), which can be island (I), peninsula (P), valley (V) or deep and hard rock (D). Table 2 lists the codes that we proposed for these criteria to be used in the DT-DNA model. For each criterion, we used a 4-alphabet code, with the first two characters representing the initials of the context type (EC).

- **Authority (A):**

The A base in the proposed DT-DNA model represents the city’s regulatory authorities. The authority can be defined as the team responsible for managing and regulating the provision of different city services and participating in decision-making processes. City council teams, city hall teams, and local health authority are examples of authorities who regulate health services in the city. When data are collected from citizens as a group, and the intended services target them on a city level, the authority who regulates the service provision must be part of the DT-DNA model of city services to guarantee the reliability of the provided services to, and the received data from, citizens through this digital twin. 

We suggest using the government type of the country and the municipality or city council to represent this base in the proposed model. Hence, we define the A base code as follows:

***Government Type-Municipality/City Council***

**Government Type:** This identifier represents the system of governance in the country. We proposed a 2-alphabet code for each government type similar to the internationally recognized TLDs of countries worldwide. We referred to the list of basic types of government and their definitions in the World Factbook [36]. According to [36], more than one government type applies for some countries, such that this identifier could be represented by one, or multiple, 2-alphabet code(s). To maintain data integrity, we add the 2-alphabet code “NN” (representing null) to country codes that have one government type when comparing with countries that have multiple government types (i.e., those with multiple 2-alphabet codes) to guarantee the fixed-location characteristic in the proposed DT-DNA model, as discussed above.

**Municipality/City Council:** This identifier represents the organization responsible for managing services in the city. Municipality (MP), city council (CC) or city halls (CH) are examples of 2-alphabet codes for authority in the city.

- **Task (T):**

The T base in the proposed DT-DNA model represents different tasks included in the provided data to represent the service required to model its DT-DNA. Since we adopted ISO 37120 [10], different indicators under each city service represent the tasks required to model the DT-DNA of each service, and to evaluate its status. Using the ISO 37120 standard indicators, our proposed code format (depicted below) requires a core (C) or supportive (S) indicator type, followed by a service initial, followed by an indicator’s clause initials.

For example, the proposed code for a service with the indicator type (C) for core, and the service initial (H) for health, and the indicator’s clause initials (ALE) for average life expectancy would be CHALE: 
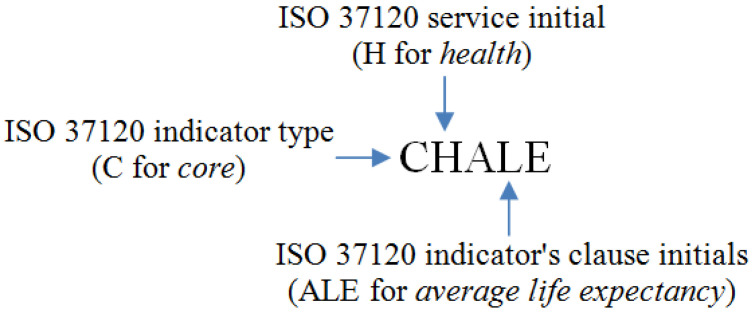


### 4.2. DT-DNA-Based Framework to Build DT of City Service

**Figure 2:**
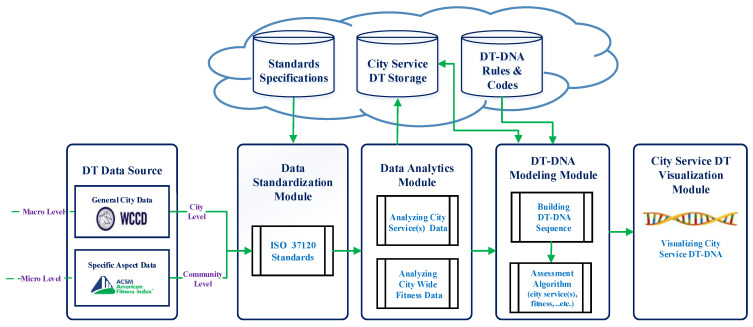
The proposed framework to build digital twins (DTs) of city services.

Figure 2 illustrates the proposed DT-DNA-based framework to build DTs of city services based on our DT-DNA model. This framework consists of the following main components (which are also illustrated in Figure 2):

- **DT Data Source:**

This component represents various sources of data for city services DTs, which are collected over time. The proposed framework addresses modeling digital twins of city services at two different levels: macro and micro. At the macro level, a city services digital twin represents the service that utilizes data collected at the city level, standardized according to the ISO 37120 standard, such as data representing health service status in the city. At the micro level, a city services digital twin represents the service that utilizes data collected from a particular community or group of citizens, also standardized according to ISO 37120, such as physically active community, diabetic community, etc. Data from a Fitness Index developed by the American College of Sport Medicines [37], which represents the fitness level of the city based on data collected from a group of citizens, is an example of micro-level data. In this level, collected data reflect responses from a group of citizens, through traditional methods such as interviews, questionnaires and surveys. The collected data in both levels are numerical data that represent the achieved percentage for each indicator in the standard.

- **Data Standardization Module:**

This component is responsible for standardizing the received data according to internationally recognized ISO 37120 standards, to guarantee the interoperability of the developed digital twins of city services. For data collected on both levels, we emphasize the importance of standardization to develop a unified general model of city services DTs that guarantees interoperability, since each city has its own way of collecting and handling data provided and used by different services. In this module, an ISO 37120-based wrapper is integrated to process the non-standardized data and map them to the proper service using standard specifications stored in the cloud.

- **Data Analytics Module:**

Once data have been standardized according to ISO 37120 [11], the Data Analytics Module is where the analysis of city services data takes place. Specific city services data or e.g., city-wide fitness data can be examined in this component. In case of big data, analysis using machine and deep learning occurs in this module. The analysis results are then sent to the DT-DNA Modeling Module for DT-DNA sequence building. The Data Analytics Module also allows for analysis results to be placed in City Service DT Storage in the cloud until they are ready for sequence building in the next module. The Data Analytics Module also allows for analysis results to be placed in City Service DT Storage in the cloud until they are ready for sequence building in the next module.

- **DT-DNA Modeling Module:**

This component is responsible for building the DNA of the city services DT, based on the proposed model in Section 4.1. When the DT-DNA Modeling Module receives data from the Data Analytics Module directly, or retrieves data from City Service DT Storage in the cloud, the module begins processing each indicator following the Task (T) naming conventions discussed in Section 4.1, and mapping each indicator to its numeric value. Other data (bases A, G, and C) are determined according to the city represented by the received data, and using the rules and codes retrieved from the DT-DNA Rules and Codes storage in the cloud. Then, the numeric values under all bases are coded to alphabetical values using a 2-alphabet code for numbers from 0 to 100, which is also stored in the DT-DNA Rules and Codes Storage in the cloud. Using the built DNA sequences, assessment algorithm of e.g., specific city services or annual fitness levels, applies through the comparison of DT-DNAs. Comparison between multiple city service DTs on different levels by comparing their DNAs and extending the DNA sequence of a particular city service DT when receiving new data are other examples of assessment algorithms occurring in this component. DNA alignment tools can be used with generated DNA sequences of city services DTs for comparison purposes such as MatGAT (Matrix Global Alignment Tool) [38]. The comparison is conducted on the newly-generated DT-DNAs or on the stored ones which are retrieved from the city service DT storage. The algorithm we propose in Section 6, called: “Which city has better services towards enhancing QoL?” is an example of suggested assessment algorithms in this module.

- **City Service DT Visualization Module:**

**Figure 3:**
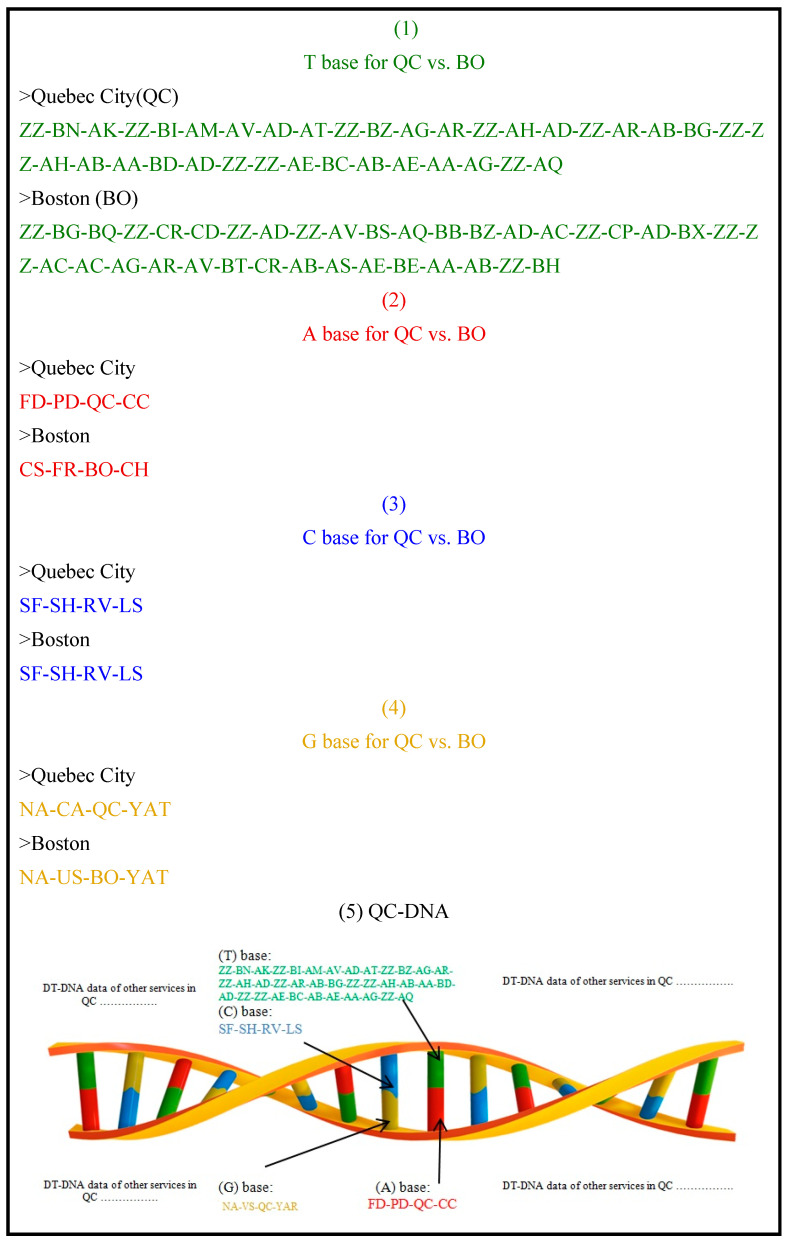

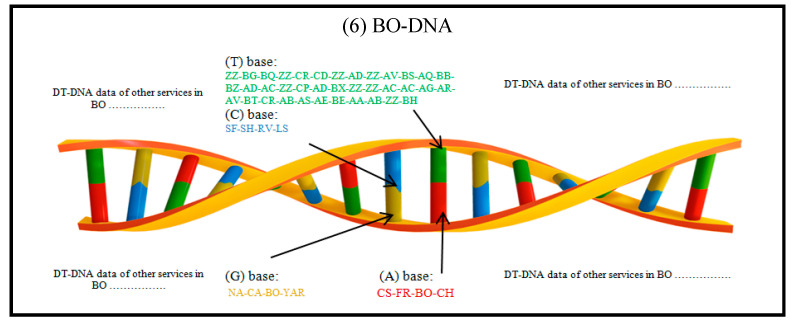
A, T, C and G bases, values of the proposed DT-DNA for QC and BO and results visualized in DNA sequences.

This component is responsible for visualizing the developed city services DTs through graphic representations of the generated DNA as shown in Figure 3 (5) and (6). This visualization can help city authorities and stakeholders gain more insight into city services, to assist them in their decision-making process with the goal of enhancing quality of life. 

## 5. Proof-of-Concept

In this section, we present the proof-of-concept of the proposed DT-DNA model and city services digital twin framework using data collected on a macro level. The proof-of-concept shows a case where the input data to the framework are standardized according to ISO 37120 [11]. We demonstrate how the proposed DT-DNA model provides results that are comparable in their accuracy to the benchmark data available, as described below. Thus, this case study shows the potential applicability of the proposed model to real-world cities. 

For the proof-of-concept, we collected benchmark data from the World Council on City Data (WCCD) portal [39], which provides data from ISO 37120-certified cities. This data was passed to the Data Analytics Module directly. Since city data in the WCCD portal [39] are presented on a yearly basis and are not available for all targeted services each year, we used data from six city services that met our benchmark criteria in the areas of health, economy, environment, transportation, recreation, and urban planning. We identified 2017 as the year that had the most available data for two cities, Quebec City (QC) and Boston (BO), and gathered data from the portal for indicators of each selected service, as shown in Table 3. 

Following the naming scheme discussed in Section 4.1, for all indicators under the six services, we named each indicator according to the suggested scheme and mapped each indicator data to its coded value as shown in Table 3. In detail, the first column shows the indicator’s clause and we bold the indicator’s initials that are used in the indicator code. The second column in Table 3 shows the indicator codes, which start with (C) for core indicators and (S) for supportive indicators. The second letter in all indicators codes represents the ISO 37120 service initial (e.g., H for Health). The last three letters in each indicator code represent the indicator’s clause initials. The third and fourth columns represent the indicator’s value for QC and BO respectively. Each value, which represents the achieved percentage for each indicator in the standard, is shown as numeric value and coded value. 

The aforementioned data represent the T base in the intended DT-DNA of QC and BO, as shown in Figure 3 (1). We removed the indicator names from the DNA sequence of each city because they are identical in each sequence and assigned a fixed position for each indicator under each service. Thus, each 2-alphabet code represents one indicator in a sequential order, separated by a hyphen from the next indicator value. Other bases will follow the same positioning rules. 

Using the city’s name, we determined A, C and G bases data utilizing stored data and web resources. The government in Canada (CA) where QC is located is a federal (FD) parliamentary democracy (PD), and QC is managed by Quebec City Council (CC). Thus, the A base of QC is represented by this sequence: FD-PD-QC-CC. Similarly, the government in the United States (US) where BO is located is a constitutional (CS) federal republic (FR) and BO is managed by Boston City Hall (CH). Thus, the A base of BO is represented by this sequence: CS-FR-BO-CH. The A base sequences are shown in Figure 3 (2). 

QC and BO are fairly identical in their C base data since both cities have flat (SF) and hilly (SH) surfaces, are built on valleys (RV) and are coastal cities (LS). Thus, the sequence of C base, particularly EC, based on ISO 37120 [11] is: ECSF, ECSH, ECRV, ECLS, as shown in Figure 3 (3). We removed the indicator’s initial from the names because it is the same in each sequence, but we assigned a fixed position for each indicator of EC.

The G base data of both cities is shown in Figure 3 (4) according to model description in Section 4.1. The DT-DNA sequences of QC and BO are shown in Figure 3 (5) and (6) respectively. 

Aligning DNA sequences in MatGAT, a DNA alignment tool used to determine the percentage of identity between two or more DNA sequences, took place at this point. Feeding QC DT-DNA and BO DT-DNA sequences into MatGAT provides 46.5% as the identity percentage of the performance level of the six services listed in Table 3, after aligning the DNA sequences of both cities. Since they are approximately half identical, this implies that one of the cities is better than the other city as shown in the case study in the following section. Comparison of our benchmark data as ground truth provides almost the same result (49.5%). 

## 6. Case Study

In this case study, we proposed an algorithm to compare cities in order to test which city has better services towards enhancing QoL based on the proposed DT-DNA. 

### 6.1. Proposed Algorithm

We proposed this algorithm (Algorithm 1) as a decomposition of the assessment algorithm under the DT-DNA Modeling Module to compare the performance of the six different services in QC vs. BO, with the goal of enhancing QoL in these cities using their DT-DNAs. We decided to compare these two cities due to the data availability limitation discussed above in Section 5, and the similar environmental context (EC) of both cities. Thus, we took both the similarity in the EC and the availability of the data for the two cities in 2017 into account when we conducted the comparison, based on ISO-standard-defined services. 

The algorithm includes assigning a score to each city based on service indicators (T) values. We developed the algorithm so that it utilizes the built DT-DNA of each city. We compared each indicator value (IV) for two given cities X and Y. If it is a core (C) indicator, and X is better than Y, the comparison value (CV) is assigned the value (+2), whereas, it is assigned the value (–2) if X is worse than Y. In a case where the indicator is supportive (S), we apply the same process, but the assigned CV is +1 or –1 respectively. Our reasoning here is the fact that the core indicator is much more important than the supportive indicator, according to ISO 37120. As there is no assigned weight for either in ISO 37120, we work with the assumption that the core indicator has double the weight of the supportive one in its importance and assigned (+/–2) and (+/–1) weights respectively. In the equal case, where X and Y have the same IV for a specific indicator, the CV is assigned the value (0). After comparing all IVs under a specific service, the sum of the CVs is calculated, and the resulting value (RV) reflects the status of X vs. Y. Positive RV means X is better than Y whereas negative RV means the opposite. The RV also shows how big the difference between X and Y is. The bigger the RV value, the bigger the difference and vice versa. The pseudo code of this algorithm is shown below.
**Algorithm 1** Which city has better services towards enhancing QoL?
read X = first city, Y = second city, E = Service under comparison;  read N = total number of indicators under E, S = total number of E in the comparison;  set I = indicator under investigation, Type = core OR supportive,    IVx = indicator value of first city, set IVy = indicator value of second city, CVi = (comparison value of indicator i) = 0, RVe = (resulted value of service E) = 0, n = 0, s = 0;  while s < S do    for each E in S do     while n < N do      for each I in E do        compare IVx AND IVy      if IVx > IVy AND Type = core then           set CVi = +2;      else if  IVx < IVy AND Type = core then           set CVi = −2;            else if IVx > IVy AND Type = supportive then           set CVi = +1;      else if  IVx < IVy AND Type = supportive then           set CVi = −1;             else // IVx = Ivy                      set CVi = 0;      end for      n++;      RVe+ = CVi;     end while     if RVe > 0 then             X is better than Y in E     else if RVe < 0 then         X is worse than Y in E     else             X AND Y are equally in E    end for    s++;    RV+ = RVe;  end while     if RV > 0 then             X is better than Y in this case     else if V < 0 then         X is worse than Y in this case        else             X AND Y are equally in this case

### 6.2. Results and Discussion

Comparison results are shown in Table 3, where EQ means equal, GT means greater than (better) and LT means less than (worse). The results show that BO is better than QC using services data collected from WCCD and the proposed algorithm, since RV = −11 when we compare QC vs. BO. 

The proof-of-concept shows promising results in terms of applicability of the proposed DT-DNA model and framework in handling city services data collected through various sources, and presenting them in a standardized, unique general model representing the city services DT. The results of applying the algorithm demonstrate its potential to compare the performance of city services towards enhancing quality of life for citizens. Results also reflect the benefit of analogy with a biological DNA model since the proposed DT-DNA model can represent any city yet protect its unique identity. The model also fulfills the entity-inclusion requirement from the human perspective through its A base for authority, and provides the means to represent fundamental as well as customized services for each city while adhering to ISO 37120 standards. The results are visualized as DNA sequences, and thus they provide an interactive tool, which enables insights into past, present and future states of a city through various services on a yearly basis. The proposed model is able to handle all 17 services listed in the ISO 37120 standards and provides a chance to accommodate future services through available chromosomes in the smart city cell. 

## 7. Conclusions and Future Work

In this paper, we suggested leveraging digital twins technology for city services, due to its potential to enhance quality of life in smart cities. We proposed a DT-DNA model where we designed a DNA of a city services’ digital twin, based on the ISO 37120 standard, reflecting the real state of the city’s development and services performance. This model guarantees interoperability, and allows for easy comparison of service performance in different cities, informing us of where a city stands when compared with other cities in different domains. We also provided a proof-of-concept for the applicability of the proposed model and the validity of the proposed codes using MatGAT (a DNA alignment tool).

As future work, we aim to build the DT-DNA of city services when the input data to the framework are not standardized according to ISO 37120 [11] in order to show how the proposed city services digital twin framework enables combining city data collected from different sources in one unique city services DT-DNA. Thus, we can extend or compare the progress of a city on a yearly basis through comparing DT-DNAs of its services, or compare the DT-DNA with DNAs of other cities in the same year towards enhancing QoL. We also plan to move towards utilizing a wider range of city data, particularly health data that are collected at the community level, and apply the proposed model to them with the goal of modeling comprehensive city services DTs. We also plan to extend the module to include federal inputs to enable modeling country DT-DNA taking into account the population number, the country’s memberships in the international forums and unions such as G20 and European Union, and the country’s position in the internationally recognized indexes such as GDP per capita and happiness index. We also plan to extend the entity-inclusion by taking non-living entities into account under the A base such as physical entities in the IoT since the current proof of concept cover the living entities or humans under the A base. We also plan to investigate the efficiency and limitations of applying the model to compare different-size cities, for example, can a small city with 100,000 people population compare effectively with cities with a few million people using the proposed model? We also plan to investigate the usage of built health DT-DNAs towards improving the quality of life in the chosen city and provide automation tools that city heads can use to compare their city with others. 

With respect to visualization of the results, we plan to conduct a study to investigate the usability of visualized DT-DNAs illustrated in the case studies, and evaluate the extent to which this visualization provides a meaningful way for citizens, stakeholders and regulatory authorities to understand and interpret results. Consequently, we can infer how people’s well-being in the cities respond to the DT-DNA analysis and compare the resulting pattern with the available patterns for health authorities.

## Figures and Tables

**Table 1:** List of all continents and codes to be used in the G base.

Continent	Code
Asia	AS
Africa	AF
Europe	EU
North America	NA
South America	SA
Oceania	OC
Antarctica	AN

**Table 2:** Environmental context criteria according to ISO 37120 [11] to be used in the DT-DNA model of smart cities.

Environmental Context (EC) Criterion	Proposed Code
**S**urface-**f**lat	ECSF
**S**urface-**h**illy	ECSH
**L**ocation-s**e**a	ECLE
**L**ocation-i**n**land	ECLN
Kind of **r**ock it is built on-**i**slands	ECRI
Kind of **r**ock it is built on-**p**eninsulas	ECRP
Kind of **r**ock it is built on-**v**alleys	ECRV
Kind of **r**ock it is built on-**d**eep and hard rock	ECRD

**Table 3:** Coded indicators, their numerical and coded values, and results of applying algorithm 1 on (Quebec City (QC) vs. Boston (BO)) data.

**Health (H)**	**Indicator code**	**QC**		**BO**		**QC vs. BO**	**−1**
**A**verage **l**ife **e**xpectancy (core indicator)	CHALE	0	ZZ	0	ZZ	EQ	0
**N**umber of i**n**-patient hospital **b**eds per 100,000 population (core indicator)	CHNNB	36	BN	30	BG	GT	2
**N**umber of **ph**ysicians per 100,000 population (core indicator)	CHNPH	10	AK	38	BQ	LT	−2
**U**nder age **f**ive **m**ortality per 1000 live births (core indicator)	CHUFM	0	ZZ	0	ZZ	EQ	0
**N**umber of **n**ursing and **m**idwifery personnel per 100,000 population (supportive indicator)	SHNNM	32	BI	62	CR	LT	−1
**N**umber of **m**ental health **p**ractitioners per 100,000 population (supportive indicator)	SHNMP	12	AM	50	CD	LT	−1
**S**uicide **r**ate **p**er 100,000 population (supportive indicator)	SHSRP	19	AV	0	ZZ	GT	1
**Economy (E)**	**Indicator code**	**QC**		**BO**		**QC vs. BO**	**−** **2**
**C**ity’s **u**nemployment **r**ate (core indicator)	CECUR	4	AD	4	AD	EQ	0
Assessed **v**alue of **c**ommercial and **i**ndustrial properties as a percentage of total assessed value of all properties (core indicator)	CEVCI	18	AT	0	ZZ	GT	2
**P**ercentage of **c**ity population living in **p**overty (core indicator)	CEPCP	0	ZZ	19	AV	LT	−2
**P**ercentage of persons in **f**ull-time **e**mployment (supportive indicator)	SEPFE	46	BZ	40	BS	GT	1
**Y**outh **u**nemployment **r**ate (supportive indicator)	SEYUR	8	AG	15	AQ	LT	−1
**N**umber of **b**usinesses **p**er 100,000 population (supportive indicator)	SENBP	16	AR	25	BB	LT	−1
**N**umber of new pa**t**ents **p**er 100,000 population per year (supportive indicator)	SENTP	0	ZZ	46	BZ	LT	−1
**Environment (N)**	**Indicator code**	**QC**		**BO**		**QC vs. BO**	**−** **1**
Fine Particulate Matter (**PM** 2.5) Concentration (core indicator)	CNFPM	8	AH	4	AD	GT	2
Particulate Matter (**PM**10) Concentration (core indicator)	CNPMC	4	AD	3	AC	GT	2
**G**reenhouse **g**as **e**missions measured in tons per capita (core indicator)	CNGGE	0	ZZ	0	ZZ	EQ	0
**NO**_2_ (nitrogen dioxide) **c**oncentration (supportive indicator)	SNNOC	16	AR	60	CP	LT	−1
**SO**_2_ (sulphur dioxide) **c**oncentration (supportive indicator)	SNSOC	2	AB	4	AD	LT	−1
O3 (**Oz**one) **c**oncentration (supportive indicator)	SNOZC	30	BG	44	BX	LT	−1
**N**oi**s**e **P**ollution (supportive indicator)	SNNSP	0	ZZ	0	ZZ	EQ	−1
**P**ercentage **c**hange in number of **n**ative species (supportive indicator)	SNPCN	0	ZZ	0	ZZ	EQ	−1
**Transportation (T)**	**Indicator code**	**QC**		**BO**		**QC vs. BO**	**−** **7**
**K**ilometers of **h**igh capacity **p**ublic transport system per 100,000 population (core indicator)	CTKHP	1	AA	7	AG	LT	−2
**K**ilometers of **l**ight **p**assenger transport system per 100,000 population (core indicator)	CTKLP	27	BD	16	AR	GT	2
**A**nnual number of **p**ublic transport **t**rips per capita (core indicator)	CTAPT	4	AD	19	AV	LT	−2
**N**umber of **p**ersonal **a**utomobiles per capita (core indicator)	CTNPA	0	ZZ	41	BT	LT	−2
**M**odal **s**plit (**p**ercentage of commuters using a travel mode to work other than a personal Vehicle) (supportive indicator)	STMSP	0	ZZ	62	CR	LT	−1
**N**umber of **t**wo-**w**heel motorized vehicles per capita (supportive indicator)	STNTW	5	AE	2	AB	GT	1
**K**ilometers of **b**icycle **p**aths and lanes per 100,000 population (supportive indicator)	STKBP	26	BC	17	AS	GT	1
**T**ransportation **f**atalities per 100,000 **p**opulation (supportive indicator)	STTFP	2	AB	5	AE	LT	−2
**Recreation (R)**	**Indicator code**	**QC**		**BO**		**QC vs. BO**	**0**
Square **m**eters of **p**ublic **i**ndoor recreation space per capita (supportive indicator)	SRMPI	8	AH	3	AC	GT	1
Square **m**eters of **p**ublic **o**utdoor recreation space per capita (supportive indicator)	SRMPO	2	AB	3	AC	LT	−1
**Urban planning (U)**	**Indicator code**	**QC**		**BO**		**QC vs. BO**	**0**
**G**reen **a**rea (hectares) per 100,000 **p**opulation (core indicator)	CUGAP	1	AA	1	AA	EQ	0
**A**nnual number of **t**rees **p**lanted per 100,000 population (supportive indicator)	SUATP	7	AG	2	AB	GT	1
**A**real size of **i**nformal **s**ettlements as a percentage of city area (supportive indicator)	SUAIS	0	ZZ	0	ZZ	EQ	0
**J**obs/**h**ousing **r**atio (supportive indicator)	SUJHR	15	AQ	31	BH	LT	−1
	**TOTAL**					**QC vs. BO**	**−11**
